# Computational and biophysical determination of pathogenicity of variants of unknown significance in cardiac thin filament

**DOI:** 10.1172/jci.insight.154350

**Published:** 2021-12-08

**Authors:** Allison B. Mason, Melissa L. Lynn, Anthony P. Baldo, Andrea E. Deranek, Jil C. Tardiff, Steven D. Schwartz

**Affiliations:** 1 Department of Chemistry and Biochemistry and; 2 Department of Biomedical Engineering, University of Arizona, Tucson, Arizona, USA.

**Keywords:** Cardiology, Cardiovascular disease, Genetic diseases, Molecular diagnosis

## Abstract

Point mutations within sarcomeric proteins have been associated with altered function and cardiomyopathy development. Difficulties remain, however, in establishing the pathogenic potential of individual mutations, often limiting the use of genotype in management of affected families. To directly address this challenge, we utilized our all-atom computational model of the human full cardiac thin filament (CTF) to predict how sequence substitutions in CTF proteins might affect structure and dynamics on an atomistic level. Utilizing molecular dynamics calculations, we simulated 21 well-defined genetic pathogenic cardiac troponin T and tropomyosin variants to establish a baseline of pathogenic changes induced in computational observables. Computational results were verified via differential scanning calorimetry on a subset of variants to develop an experimental correlation. Calculations were performed on 9 independent variants of unknown significance (VUS), and results were compared with pathogenic variants to identify high-resolution pathogenic signatures. Results for VUS were compared with the baseline set to determine induced structural and dynamic changes, and potential variant reclassifications were proposed. This unbiased, high-resolution computational methodology can provide unique structural and dynamic information that can be incorporated into existing analyses to facilitate classification both for de novo variants and those where established approaches have provided conflicting information.

## Introduction

Familial cardiomyopathies often result from single point mutations within the genes encoding the proteins of the cardiac sarcomere, the primary functional contractile unit in striated muscle. The 2 most commonly observed forms of these cardiomyopathies, hypertrophic (HCM) and dilated (DCM), are clinically characterized by complex, progressive, and largely divergent patterns of left ventricular remodeling (1, 2). While the original reports detailing the clinical expression of HCM included familial cases, initial genetic linkage studies were first published in 1990 (*MHY7*, encoding the myosin heavy chain), closely followed by components of the cardiac thin filament (CTF) and myosin binding protein C (3, 4). Recent advances in low-cost, high-throughput sequencing of genomes and exomes have increased testing and diagnostic sensitivity, with a concomitant increase in the identification of variants of unknown significance (VUS; ref. 5). Recently, a 5-tier system of variant classification has been recommended using the base term “variant” with either pathogenic, likely pathogenic, uncertain significance, likely benign, or benign modifiers appended (6, 7).

A significant proportion of VUS are located in the genes encoding the CTF, including *TNNT2* and *TPM1* (8). The CTF is composed of 3 protein complexes, tropomyosin (Tm), troponin (cTn), and actin, that act together to activate muscle contraction via the allosteric binding of calcium to site II of cardiac troponin C (cTnC; ref. 9). Tm blocks myosin binding sites on the actin surface until calcium binds to cTnC, causing conformational changes within the CTF to allow Tm to traverse the actin surface, revealing the binding sites for myosin association. This process, originally proposed by Geeves and McKillop, is referred to as the three-state model of myofilament activation (10, 11).

Numerous studies have probed effects of mutations within the CTF with respect to dynamics and structure of the sarcomere (4, 12–18). Despite these extensive efforts, many of the fundamental, primary mechanisms that lead to the complex, progressive pathogenic cardiac remodeling that defines the clinical disorder remain elusive (19). Enduring challenges remain, including the ability of mutations in single components of the multiprotein complex to affect myofilament function in distant functional units and the difficulty in linking primary structural and dynamic molecular perturbations to morphological manifestations of a progressive cardiomyopathy. To address the molecular component of this challenge, we have previously developed an atomistic model of the CTF that includes all protein components and regions. To our knowledge, this is the only full atomistic model and is critically important as one addresses mutations near “unstructured” regions not resolved in any previous experiment, where most variants arise (20). The full atomistic model has previously been used as a mechanistic probe to simulate structural and dynamic changes induced by mutations that are not addressable by experiment. These studies have revealed that point mutations in the CTF can result in structural and dynamic changes propagated over hundreds of angstroms (4, 9, 21–23). While elucidating the primary mechanisms underlying pathogenic remodeling is crucial, from the clinical standpoint, rigorous assignment of pathogenic potential to de novo variants or those where existing information is conflicting has emerged as a central challenge to the use of genetic information in the care of families with HCM (7).

In the current study, we now apply our all-atom computational model of the CTF coupled to biophysical metrics of structure and dynamics to develop a robust, nonbiased approach to classifying HCM VUS as benign, likely benign, likely pathogenic, or pathogenic. A set of 15 well-defined pathogenic variants on cardiac troponin T (cTnT) and 6 pathogenic variants on Tm were simulated using the fully atomistic model previously created by our group to determine a “baseline” of primary structural and dynamic effects. Molecular dynamics simulations were run on 9 cTnT and Tm VUS, and results were compared with the baseline pathogenic behavior previously determined. Variants that exhibited similar metrics in simulations as well-defined pathogenic mutations were expected to be pathogenic or likely pathogenic while variants that rendered significantly different signatures than pathogenic mutations were expected to be benign or likely benign. It is important to note that while this new, unbiased addition to current metrics in use to assess VUS pathogenicity is meant to be additive and not proposed to be the “sole” determinant of classification, we conclude that this approach can play a potentially important role in adding to the rigor of this complex, and highly clinically relevant, multimodal assessment.

## Results

Molecular dynamics (MD) simulations are an invaluable method to investigate protein conformations, dynamics, and thermodynamic data. These approaches are essential for detecting high-resolution changes in biological systems, such as proteins, that are not readily accessible by experimentation. MD allows all atoms within a model to interact for a fixed period, providing visualization of the dynamic motion of the system. Initial placement of atoms is determined from published structures in the protein data bank. MD then allows the location at a later time and the movement of atoms over time to be determined. This motion is obtained by numerically solving Newton’s equations of motion, where forces between particles and potential energies are calculated using molecular mechanics force fields. Once locations and movement over a fixed period have been obtained, data analysis can be performed to extract important atomistic-level details, such as distances between proteins. Since distances from different regions will all be on different scales, they cannot be directly compared to each other during analysis. Therefore, standardization of distances (*z* scores) is needed, which allows for the direct comparison of variables that are on different scales (24).

Using the full atomistic model our group previously created to probe structural and dynamic changes due to mutations linked to HCM, we have evolved the use of computations in this report to develop an unbiased approach to classify de novo or reclassify indeterminate VUS based on the possession of similar structural characteristics to established pathogenic CTF mutations. To determine a baseline of pathogenic structural changes in the CTF, we constructed a list of 21 independent and highly curated HCM-linked variants that cause pathogenic missense mutations in Tm and cTnT, the protein linking Tm to the cTn complex. The mutations and their locations on the CTF are grouped according to the protein in which they occur as well as their location on the CTF, as shown in Table 1.

**Figure 1:**
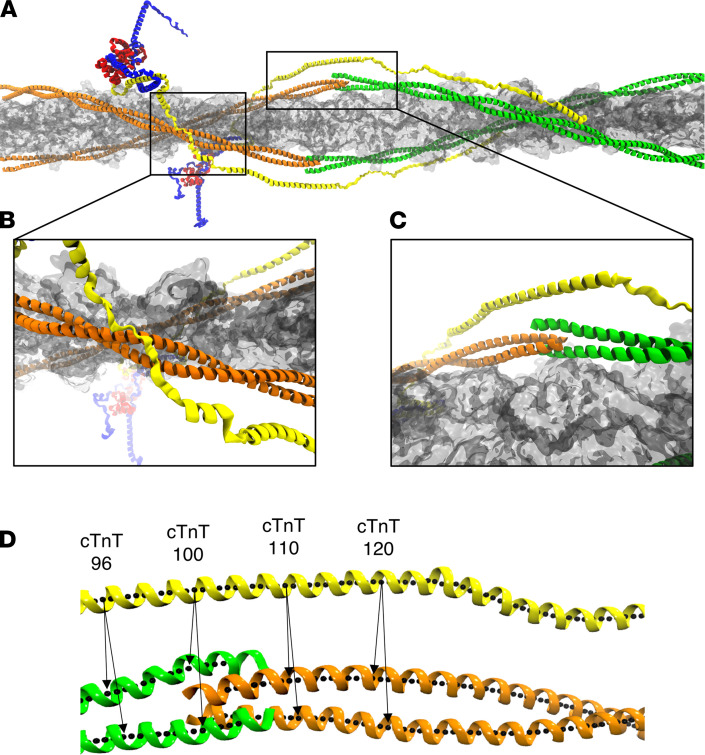
The complete model of the CTF. (**A**) Actin (gray), Tm (orange and green), cTnC (red), cTnI (blue), and cTnT (yellow). The zoomed-in view of region B (**B**) containing Tm (residues 160–230) and the unstructured linker region of cTnT (residues 155–220). The zoomed-in view of region A (**C**) containing the overlap region (cTnT 80–150; Tm 1–50, 250–284) where Tm dimers overlap in a head-to-tail fashion. (**D**) The overlap region of the CTF along with the center of mass of the respective helices (black dots) used to calculate the distances between proteins. Four specific cTnT residues are labeled, with arrows showing the closest Tm residue used to calculate the overlap distance and distribution plots.

Conformational and dynamic changes throughout the CTF were calculated for all 21 mutations to create a data set designed to provide high-resolution pathogenic signatures. Conformational changes were defined by the physical distances between cTnT and Tm in the overlap region of the CTF (cTnT 90–140; Tm 1–20, 250–284), between the N-terminal Tm dimer and directly adjacent actin monomer, and between the N-terminus Tm coils, as shown in Figure 1. Changes were calculated using center of helix distances and compared with WT results to identify trends (Supplemental Tables 1 and 2; supplemental material available online with this article; https://doi.org/10.1172/jci.insight.154350DS1). We note that these structural parameters are presented as single numbers, but they are derived from extensive MD simulations and thus represent averages of dynamic structures. We have found such averages to be sufficient for reclassifying VUS here, but the full simulation is available for further algorithmic development in the future.

The CTF was divided into 2 regions of mutations based on prior studies, the natural clustering of genetic mutations, and the secondary structure of cTnT and Tm in the respective region (20). Region A (cTnT 80–150; Tm 1–50, 250–284) contains the overlap region, which consists of fully alpha-helical secondary structures for both cTnT and Tm, while region B (cTnT 155–220, Tm 160–230) contains an “unstructured” cTnT interacting with the Tm coiled coils.

Following analysis of known pathogenic mutations, 9 individual cTnT and Tm VUS located in the same regions as the pathogenic variants analyzed were simulated using the full atomistic model of the CTF. Structural and dynamic changes were subjected to the same analysis as pathogenic mutations with respect to WT. All structural changes were standardized with respect to the local region (region A or B). Using the *z* scores, the Euclidean distance from the origin (average pathogenic value) was calculated to determine if a given VUS possessed similar or different structural characteristics from pathogenic mutations (Tables 2 and 3).

**Figure 2:**
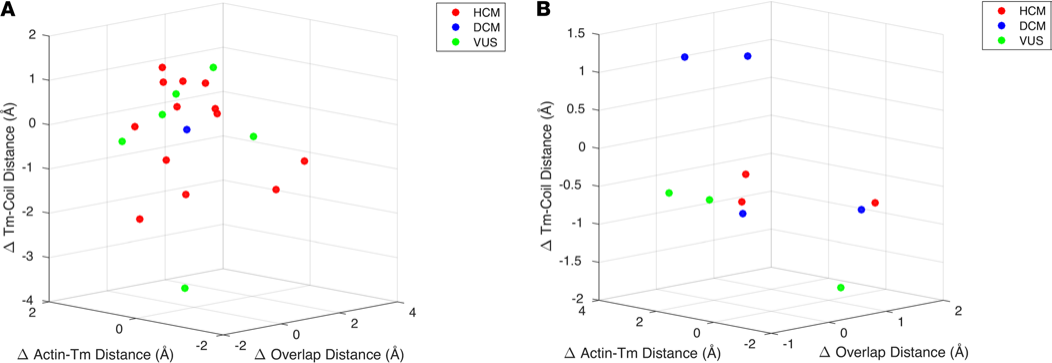
3D scatter plots of standardized structural distances calculated from MD simulations for VUS (green) and pathogenic mutations, HCM (red) and DCM (blue). All distances were calculated with respect to WT values and then standardized with respect to the region they are located in, region A (**A**) or region B (**B**).

The 3D scatter plots of the structural variable *z* scores calculated for each variant in region A and B are shown in Figure 2. The plots reveal a natural clustering of several pathogenic variants along with VUS A22T_Tm_, D28H_Tm_, A277T_Tm_, K97N_cTnT_, and A104V_cTnT_ from region A and L178F_cTnT_ from region B. The VUS M281V_Tm_ was separated from the cluster from region A. Similarly, the VUS V218L_cTnT_ in region B was separated from the cluster of pathogenic variants.

**Figure 3:**
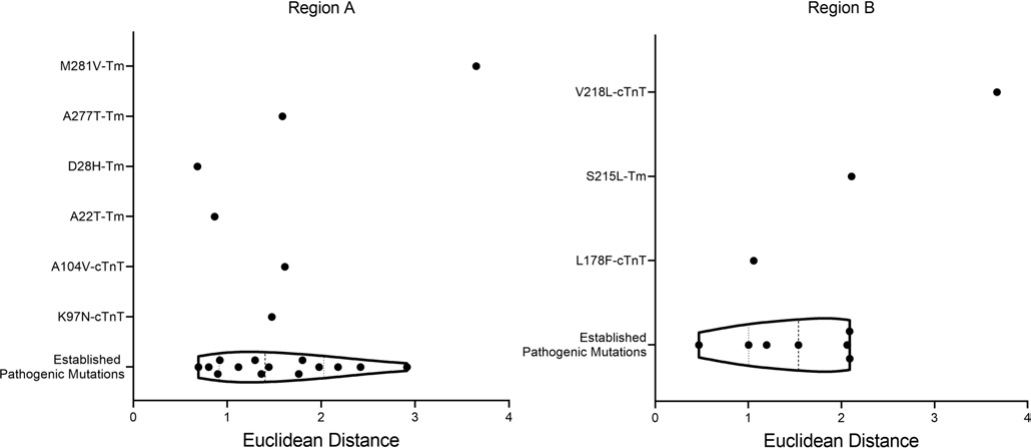
Violin plots using the Euclidean distance from the origin of standardized distances calculated in MD simulations. Mutations in regions A and B, respectively, are standardized separately. VUS are plotted separately from pathogenic mutations to determine overlap with pathogenic distribution. Data are shown with median and interquartile range (dashed black lines).

To confirm whether VUS were outliers from pathogenic mutations, the Euclidean distances from the origin were calculated (Tables 2 and 3). This allows for a direct comparison of a single number representing the distance each variant lies from the average pathogenic values. By plotting all pathogenic variants within each region together, the distribution of variants in a given region is revealed (Figure 3). All pathogenic mutations have been clustered together regardless of specific phenotype. Therefore, predictions proposed in this manuscript are focused on determining whether VUS are predicted to be pathogenic, not what the morphological development may look like. The interquartile range (IQR) represents the middle 50% of pathogenic mutations. Therefore, VUS that lie in this region are predicted to be pathogenic. Any VUS that fall outside the IQR but still align with pathogenic variants (upper and lower 25%) would be reclassified as likely pathogenic. Any VUS that fall outside the boundaries of the pathogenic distribution, but are not statistically considered outliers compared to pathogenic variants, are predicted to be likely benign, while those that are statistical outliers from the pathogenic boundaries are proposed to be reclassified as benign. Outliers are defined as variants that are farther than 1.5 × IQR above the upper quartile or below the lower quartile (Q3 + [1.5 × IQR] or Q1 – [1.5 × IQR]).

### Differential scanning calorimetry.

While MD simulations can be used alone to recategorize VUS as benign/likely benign/likely pathogenic/pathogenic, it is critical to establish that this observation can be attributed to experimentally verifiable predictions of structure and dynamics of the CTF. Thus, to verify the computational results, differential scanning calorimetry (DSC) using fully reconstituted thin filament proteins containing cTnT, cardiac troponin I (cTnI), cTnC, Tm, and actin was used to measure the heat capacity (kJ/mol × K) during unfolding of the CTF over a temperature range. Four of the analyzed variants were located in region A and in close proximity to one another, while the last VUS, S215L_Tm_, was located in region B (Table 1) yet was still relatively close to the other mutations analyzed.

**Figure 4:**
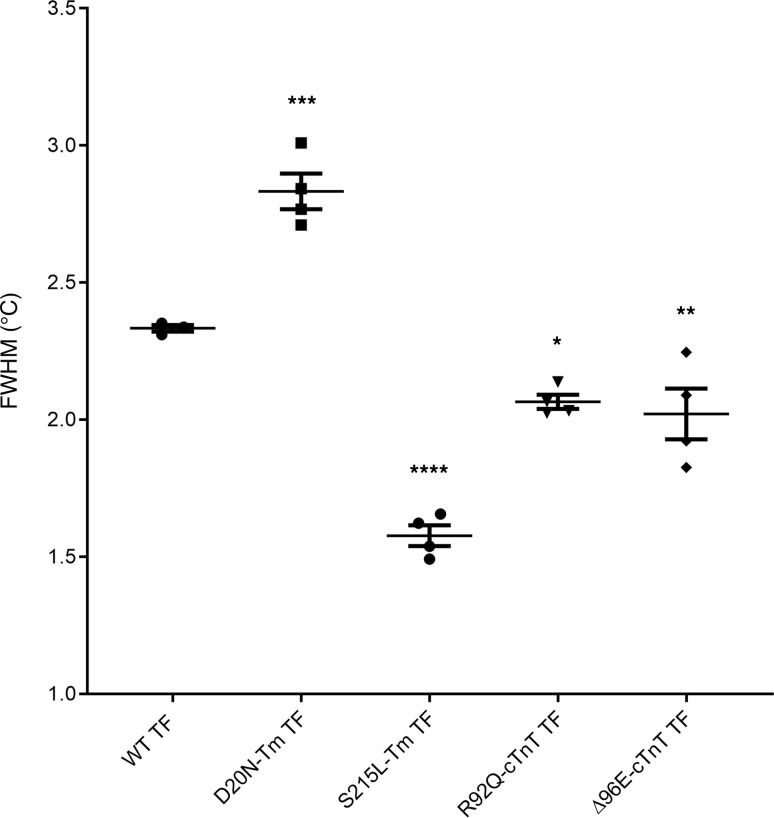
FWHM results from DSC experiments for fully reconstituted thin filaments of WT, D20N_Tm_, S215L_Tm_, R92Q_cTnT_, and Δ96E_cTnT_. Sample sizes of *n* = 4 for WT and each mutant. The error bars represent mean ± SEM. Significance assessed by 1-way ANOVA with Dunnett’s correction for multiple comparisons and defined by *P* < 0.05 (*), *P* < 0.01 (**), *P* < 0.005 (***), *P* < 0.0001 (****).

The most basic thermal unfolding processes are “two-state” such that, as temperature rises, the protein, or protein complex, transitions from the native “folded” state to the denatured “unfolded” state with a Gaussian distribution. When an increase in the full-width half-maximum (FWHM) of the transition is observed, it becomes less “two-state-like” with more intermediate complexes possible (25, 26). This, in turn, is consistent with an expected increase in conformational flexibility of the protein (or decreased cooperativity of unfolding). As the FWHM decreases, the opposite is true, suggesting a decrease in flexibility (27, 28). It is important to note here that our use of the terms cooperativity of unfolding and (inversely) conformational flexibility is established terminology used in calorimetry to describe the thermal outputs reported in our DSC (thermal stability and FWHM; refs. 9, 25–33). By “unfolding,” we are referring to broad changes in structure that may be a single protein dissociation or global complex dissociation (e.g., Tm dissociating from actin) induced by heat absorbed in the calorimeter. We and others have shown that DSC is a valuable technique for studying thin filament thermodynamics, giving regionally specific information on thermal stability and conformational flexibility of these proteins in complex (9, 29–31, 34). Here we used DSC as a method to biophysically confirm the model-derived changes in root mean square fluctuation (RMSF), a related standard measure of flexibility that calculates the fluctuation of each alpha carbon in a protein and can then be compared with the WT structure (23). Our data show that D20N_Tm_, a mutation linked to HCM, significantly increased the FWHM of the Tm-Tn array unfolding (Figure 4), consistent with an increase in conformational flexibility of the proteins in the overlap region. These experimental results agree with the MD simulations, which revealed an increase in the RMSF of cTnT (Supplemental Figure 1) and a concomitant increase in the overlap distance between cTnT and Tm compared with WT (Supplemental Table 1). R92Q and Δ96E, pathogenic variants in cTnT associated with HCM, both significantly decreased the FWHM of the Tm-Tn unfolding with respect to WT (Figure 4), indicating a decrease in flexibility and increase in cooperativity with the proteins in the overlap region. MD simulations revealed that the RMSF of R92Q in the Tm termini decreased slightly (Supplemental Figure 2), in agreement with the DSC experimental data. For Δ96E, MD data showed the average distance between actin-Tm significantly decreased (Supplemental Table 1), suggesting a compaction of the overall Tm-Tn overlap complex, which likely explains the observed decrease in flexibility. S215L, a Tm-linked VUS, exhibited a significant decrease in FWHM of the Tm-Tn array (Figure 4), indicating a decrease in conformational flexibility. MD calculations showed the average distance between actin-Tm and the average distance between Tm coils decreased, suggesting the protein assumed a more compact structure in this region, leading to a decrease in overall flexibility of the Tm-Tn overlap complex (Supplemental Table 2).

The combination of experimental and MD data strongly supports the hypothesis that mutational effects on the conformational flexibility of Tm and cTnT depend on changes apparent in both structure and dynamics. Importantly, while DSC offers regionally specific thermal information and experimental confirmation of our model-derived values, it is a cumulative measure of heat capacity in calorimetric unfolding of a reconstituted thin filament, and thus, is not precise enough to determine the exact structural change or predict resultant pathology. An advantage of combining DSC with our computational methods is that we can differentiate dominant structural factors for each individual variant that led to these cumulative changes in conformational flexibility and inform predictions of pathogenicity for independent VUS.

### cTnT and Tm variants.

To test our computational approach to assigning pathogenicity, we chose 9 independent cTnT and Tm VUS and performed MD as noted above (Table 1). These VUS were selected on the basis of a lack of definitive assignment as per ClinVar and/or found to be primarily observed in combination with other variants such that their independent pathogenic potential could not be assessed. In other words, they were not clinically actionable based on established criteria. Note that all “test” VUS were provided in a blinded fashion to the computational group.

In region A, the VUS A22T_Tm_ had a Euclidean distance of 0.865 (Table 2). This falls outside the IQR but in line with pathogenic mutations R94H_cTnT_ (0.900), F110L_cTnT_ (0.803), and L278F_Tm_ (0.920). Based on the calculations, A22T_Tm_ is suggested to be likely pathogenic, similar to the established HCM-linked mutations R94H_cTnT_, F110L_cTnT_, and L278F_Tm_. D28H_Tm_ had a Euclidean distance of 0.683 (Table 2), placing this VUS outside the pathogenic boundary regions (Figure 3). Based on these results, it was not statistically considered an outlier; therefore, D28H_Tm_ was predicted to be likely benign. A277T_Tm_ had a Euclidean distance of 1.588 (Table 2), placing it inside the IQR of the pathogenic variants plot (Figure 3). Pathogenic mutations D86A_cTnT_ (1.762) and R130C_cTnT_ (1.442) were proximal. Therefore the VUS A277T_Tm_ was predicted to be pathogenic, similar to D86A_cTnT_ and R130C_cTnT_, both HCM-causative mutations. M281V_Tm_ had a Euclidean distance of 3.650 (Table 2), making it an outlier from the pathogenic behavior of variants in region A. In addition, this variant was far removed from the cluster of pathogenic variants in Figure 2, further solidifying the prediction that this mutation does not show characteristics aligning with pathogenic mutations in MD simulations. Given this observation, the recommendation would be to reclassify M281V_Tm_ as benign. K97N_cTnT_ had a Euclidean distance of 1.475 (Table 2), which is within the IQR of the pathogenic mutations. Therefore, this VUS is suggested to be pathogenic similar to R130C_cTnT_ and R94C_cTnT_, both HCM-linked mutations that are proximal to K97N_cTnT_ in the violin plot (Figure 3). Finally, A104V_cTnT_ had a Euclidean distance of 1.614 (Table 2), falling inside the IQR (Figure 3). Due to this, we would expect A104V_cTnT_ to be pathogenic, similar to the adjacent pathogenic mutations D86A_cTnT_ and R130C_cTnT_, both established HCM-linked mutations.

In region B, the VUS S215L_Tm_ had a Euclidean distance of 2.109 (Table 3), placing it outside the region of defined pathogenic parameters for this region. Statistically, it was not sufficiently distant to be considered an outlier, and therefore we expect this variant to be likely benign. The cTnT VUS, L178F (1.058), lay within the IQR of the defined pathogenic mutations in region B. It was closest to the DCM-causative variant R173Q_cTnT_. Therefore, L178F_cTnT_ is suggested to be reclassified as pathogenic. Finally, the VUS V218L_cTnT_ had a Euclidean distance of 3.671 (Table 3). This was sufficiently distant from the defined pathogenic boundaries for region B, classifying it as an outlier. Therefore, we would suggest V218L_cTnT_ be reclassified as benign.

### Distributions.

**Figure 5:**
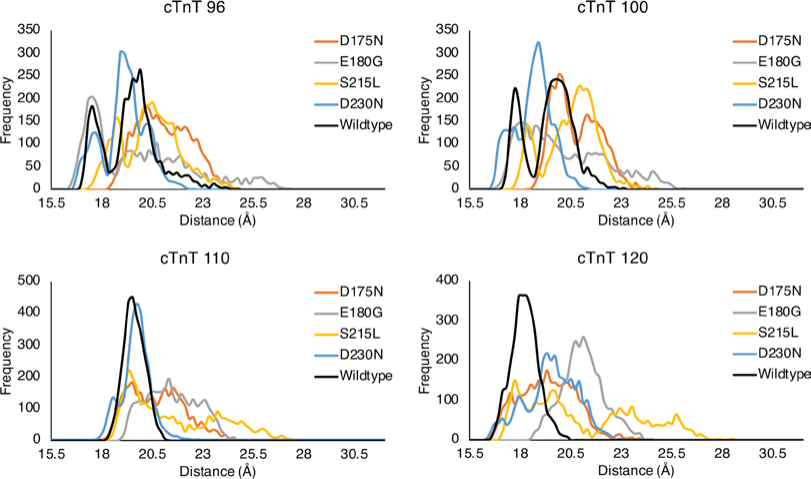
Binned distributions of distances across all trajectories between the specified cTnT residue and the 2 Tm chains for WT and all variants on Tm in region B (D175N, E180G, S215L, and D230N). These distances were incorporated in the overall average distance between cTnT and Tm in the overlap region.

The values presented in Tables 2 and 3 are clearly a composite result of subtle changes in dynamics of the overall CTF complex. To better visualize the origin of the values, one must investigate more closely the changes in data that give rise to average distance changes. In Figure 5, we show binned distributions of distances for specific residue pairs in the overlap region. Individual graphs in Figure 5 are labeled via a specific residue in cTnT and represent the distribution between that residue and the cognate closest Tm residue across all MD trajectories. In this figure it is clear that the overall distance reported is the result of a basic shift in the overall distribution of distances. In general, uni- or bimodal distributions of distances become skewed and more broadly distributed across the distance spectrum. There are no error bars on these plots because these are exact numbers, which result from integrating the equations of motion within our model. This shift in distribution was due to the crafted evolution of the WT CTF with 1 or 2 free energy minima for each cTnT-Tm residue pair, resulting in 1 or 2 favored distances with small fluctuations around these favored positions. Mutations induced a shift in the protein dynamics, which, in turn, was apparent in the basic shapes and distributions as shown. Thus, the distance values reported in Tables 2 and 3 are manifestations of mutation-induced changes in protein dynamics. Plots for all variants are included in the data supplement (Supplemental Figures 3–9).

### New model.

**Figure 6:**
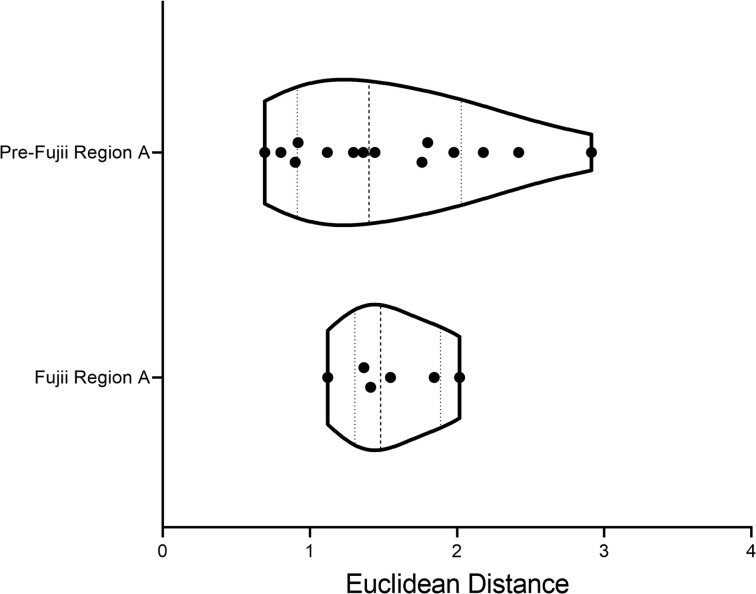
Comparison of violin plots for pathogenic variants on cTnT in region A using the pre-Fujii model (top) and the new Fujii model (bottom). Data are shown with median and IQR. There was no difference between results from the 2 models, as indicated by the medians and distribution of data. Plots were generated using Prism version 9.0.0 (GraphPad).

Recently, a new CTF structure was published and subsequently refined (35–41). To further verify the results presented here, we corrected specific errors in the Fujii structure (e.g., an incorrect helical pitch in Tm) and added our verified structure in regions unresolved in this cryo-electron microscopy (cryo-EM) approach (35). Using the new model, 6 pathogenic mutations located on cTnT in region A were used to verify the defined pathogenic behavior in this region as presented above. The mutations simulated include D86A, F87L, R92L/Q/W, and R141W (Table 4). Following the same analysis technique described above with the previous model (Supplemental Table 3), the results showed the average pathogenic characteristics were similar to those defined above (Figure 6). Plotting all pathogenic mutations from the old model and new model allowed comparison of the mean and distribution of data around the mean, which revealed no difference between the 2 models. The smaller variance in the new model is likely due to analyzing 6 variants versus the 14 variants analyzed in this region using the original model. Thus, our results remain consistent with the analysis presented herein and maintain a high level of overall accuracy, demonstrating that new structural information can easily be continuously incorporated into the methodology as it becomes available.

## Discussion

Pathogenic cTnT and Tm variants pose significant risk for significant cardiac remodeling (1, 4). Genetic testing to screen for these variants is used significantly more often. However, for an ever-expanding number of variants, understanding of pathogenicity remains insufficient. The specific molecular and organ-level mechanism(s) whereby most variants result in pathogenic or benign phenotypes are still unclear, further obscuring the ability to both treat patients and improve the care of potentially affected family members (7). Moreover, because the ability to identify patients in the preclinical state (genotype^+^, phenotype^–^) facilitates the eventual possibility of earlier treatments to change the natural history of these complex disorders, additional, rigorous approaches to further clarify the limbo of the VUS state is of significant clinical importance.

A variety of approaches have been proposed to predict potential pathogenicity of clinical variants, such as the PolyPhen-2 algorithm, the Single Amino Acid Polymorphism Data Analysis Predictor (SAAPdap) algorithm, homology modeling, etiological fractions (EFs), and support vector machines. The PolyPhen-2 algorithm uses sequence alignments and structural features of 3D proteins to predict the potential impact of amino acid substitutions (42). This algorithm was designed to be easily accessed, to be widely used, and to enable rapid predictions but cannot predict effects of splice variants, insertion or deletion mutations, as well as mutations that affect the function of neighboring proteins within a complex. Similarly, the SAAPdap method utilizes structural analysis as well as machine learning to predict the gain or loss of function due to a mutation within a protein (43). This method is highly focused on the local structural environment and therefore is more accurate than the PolyPhen-2 algorithm. However, it cannot determine alterations to structure or function long distances from the mutational site, which have been shown to be significant (9, 21, 22). Homology modeling of pre- and postpowerstroke states of myosin was utilized in an attempt to find regions of disease-causing variants (44). This highly static approach was able to identify regions of variant clusters; however, it is left unexplained how these variants affect the protein function or other conformations of myosin. Another technique, designed by Walsh et al., uses EFs to approach the prediction problem from a clinical application method (45). This technique is dependent on the location of the variant within a gene/protein and estimates the probability that the variant is pathogenic. While this was designed to be used in a clinical setting, it uses various prediction algorithms as inputs, which have been shown to have widely varying values as predictors of clinical outcomes. The authors also note that for this method to be accurate, it should only be applied to evaluating variants in people who are clinically affected. Finally, support vector machines, an artificial intelligence technique, have been utilized in an attempt to resolve the clinical prediction problem by using various phylogenetic and structural information descriptors (46). Two of the input descriptors include PolyPhen-2 and a protein structure comparison score. All of these methodologies discussed rely on a mixture of mutation position and some degree of structural information obtained from the WT proteins. None, however, directly incorporate structural changes because of mutation or how different components of the sarcomere interact. We note that the function of the three-state model requires complex allosteric interactions between the multiple components that make up the thick and thin filaments, with the effect propagating over long distances (e.g., Ca^2+^ binding; refs. 9, 21, 22). While each method has advantages, they all focus purely on local effects and are inherently static, neglecting fundamental protein dynamics and the importance of allostery.

Recently, Pettinato et al. studied the functionality of 51 cTnT variants by comparing cardiac microtissue contractions and B-type natriuretic peptide reporter activity of 30 pathogenic/likely pathogenic variants and 21 VUS (8). They found that HCM-associated variants increased contraction while DCM-associated variants decreased contraction with respect to WT controls. After finding this distinction between pathogenic variants and WT controls, they were able to reclassify 2 VUS as pathogenic/likely pathogenic. However, as noted by the authors, most human induced pluripotent stem cell cardiomyocytes used for variant modeling resemble neonatal cardiomyocytes in both expression and function. In particular, these cells express the skeletal form of troponin I (missing the first N-terminal 30 amino acids), changing a crucial component of the PKA-mediated regulation of β-adrenergic signaling at the myofilament level. Thus, while these studies provide additional potential mechanistic insight, the use of broad, binary functional measures may be limited as a classification system in a disorder defined by long-term ventricular remodeling and phenotypic variability.

The current manuscript details the ability to reclassify VUS using average structural data gathered from extensive MD simulations of an all-atom model of the CTF as a new, additive component of VUS assessment. A refinement of the current classifications was suggested for 9 genetic VUS located on cTnT and Tm based on variant location and structural effects induced on the overlap region compared with a total of 21 well-defined pathogenic mutations. DSC was used to experimentally corroborate the calculations in vitro and revealed that both structural and dynamic changes played a role in the change in cooperativity and flexibility of the proteins in the CTF. This is the first time to our knowledge that high-resolution biophysical data from the entire CTF structure has been incorporated in an effort to reclassify the pathogenicity of genetic VUS. The structural and dynamic data extracted in this study contribute valuable insight to further understand the disease states that accompany variants in the CTF.

Overall, based on analysis of MD simulation data in region A, the suggested reclassifications of VUS are as follows: M281V_Tm_ is benign, D28H_Tm_ is likely benign, A22T_Tm_ is likely pathogenic, and K97N_cTnT_, A104V_cTnT_, andA277T_Tm_ are pathogenic. Based on data from region B, the suggested reclassifications of VUS are the following: V218L_cTnT_ is benign, S215L_Tm_ is likely benign, and L178F_cTnT_ is pathogenic (Table 5). Note the proposed reclassification of S215L_Tm_ is of interest given the multiple reports of this variant, with most occurring in the context of other variants or known mutations, thus leading to considerable controversy as to whether it is an independent pathogenic allele (47, 48).

While the approach presented is rigorous and nonbiased, there are some limitations to the current study. The reclassifications presented here were based on averages of 3 variables all located in the overlap region from 3 independent 10 ns simulations. These averages are simplifications of more complex shifts in distance distributions, as shown (Figure 5). Given the previous work we have published, we acknowledge structural and dynamic changes induced by these variants in other regions of the CTF as well as changes in interaction between the thin and thick filaments. For example, changes in Ca^2+^ metrics and signaling pathways are possible sources that may lead to disease, which might not have been captured in the structural data analyzed in the current study. We also note that the 10 ns simulations have proved sufficient to accurately model mutational effects in the past. These distributions and changes are difficult for an individual to assess, but a more automated process, such as deep machine learning, is exactly tailored to such a task and is a topic of ongoing research. In addition, understanding the pathogenic effects on interactions of the CTF with other proteins in the sarcomere (e.g., the thick filament) is under intensive study (49, 50). Moreover, we hope to be able to expand this analysis to other regions of the CTF, such as the C-terminus of cTnT, and to extend the length of simulations in the future. This region, in particular, remains poorly resolved in all extant structures, including in the most recent manuscript by Risi et al. (36). In addition, identification of well-defined pathogenic mutations in the C-terminus of the CTF has lagged as there remains a high level of uncertainty regarding the structural and dynamic landscape in this highly flexible domain. Given our ability to quickly iterate the CTF with new structural information and our own efforts to clarify the structure of the C-termini of cTnT and cTnI, we are optimistic that this important mutational “bin” will be included in future studies.

The model used for all calculations presented in this paper was created using all structural data available to date. As more structural data become available, the model has been and will be fine-tuned if needed to incorporate new information. As updating classifications for variants every few years is standard procedure as more data are available and more patient screenings are performed, we propose that our approach can be incorporated into these reassessments when new structural insight is obtained.

As noted in the 2020 guidelines, at present, identification of VUS is not a clinically actionable result, and such uncertainty is the basis for the periodic reevaluation of variants (7). We propose here that our computational methodology can be incorporated into the existing assessment of novel VUS to provide unbiased molecular/functional rigor that can improve the fidelity of classification. Also note that the exclusive use of human proteins and sequences is an advantage and also overcomes some of the challenges inherent in the use of mixed-species in vitro studies in prediction, which can provide conflicting information because of variations in experimental protocols. Overall, the theme of the current work and any future work on this topic is that both structure and dynamics determine function, or dysfunction, and detailed studies of these properties may be used to inform rigorous reclassifications of pathogenicity to facilitate the use of genetic information for cascade screening and eventual management decisions.

## Methods

Each simulation using our original model started from the same low-temperature structure that has been described previously (9, 21, 22). However, the structure has been updated to utilize the CHARMM36 parameters, which encompass the latest and most accurate version of the CHARMM force field (51). Point mutations were incorporated into each corresponding CTF protein with the CHARMM42 program by deleting side chain atoms of the desired residue and rebuilding it to the desired new residue based on the CHARMM36 parameters (52). Deletion mutations were incorporated by deleting all side chain and backbone atoms for the residue of interest and directly bonding the backbone nitrogen of the previous residue to the backbone carboxyl carbon of the next residue. These methodologies have been previously described and utilized (9, 21, 23).

### Simulation parameters.

Once mutations were incorporated into the protein structures, the system was solvated with TIP3P waters using the SOLVATE plugin in VMD1.9.3 (53), with the water box extending at least 15 Å from the protein surface. Potassium and chloride ions were added randomly within the water box to a set concentration of 0.15 mol/L with the AUTOIONIZE plugin in VMD1.9.3 (53). All simulations were performed with NAMD version 2.12 with the SHAKE algorithm to constrain heavy atom-hydrogen bonds (54). Nonbonded interactions were calculated with the particle mesh Ewald method with a cutoff value of 12 Å. Each system was subjected to 5000 steps of minimization with the conjugate gradient method, then slowly heated to a temperature of 300 K at a rate of 1 K/ps. Finally, the system was equilibrated in an isobaric-isothermal ensemble at 1 atm and 300 K for 690 ps using a Langevin piston Nosé-Hoover method to control the barostat and Langevin dynamics to control the thermostat. From the equilibrated structure, 3 independent 10 ns production runs were performed by randomly generating velocities from a Boltzmann distribution. From these production runs, all statistics were measured and determined.

### Structure and dynamics characterization.

Average overlap distances were assessed by calculating the distance between the center of mass of the cTnT helix, residues 90 to 140, with the center of mass of the N-terminal and C-terminal Tm coiled coils throughout the production runs. Changes in distance between the N-terminal Tm coils were calculated using the distance between the center of mass of the helices, residues 1 to 20, averaged throughout the production runs. Changes in distance between Tm and actin were calculated between the center of mass of the N-terminal Tm coil and neighboring actin monomer located directly below. Changes in flexibility were determined by measuring the RMSF of the alpha carbons in the proteins for each simulation. This equates to determining the SD in the atom’s position with respect to its average position.

### 3D scatter plots, violin plots, and distribution plots.

The *z* scores for each variable were plotted on a 3D scatter plot using Matlab to visualize natural clustering of pathogenic mutations and VUS (55). Euclidean distances were used to generate violin plots to determine distribution of data using Prism version 9.0.0 (GraphPad). Distribution plots were created by binning individual distances across trajectories for each variant and the WT structure. Plots were labeled with a specific cTnT residue indicating the distribution of the distance between the given cTnT residue and closest residue on the 2 Tm chains across all trajectories. Bins with a width of 0.2 Å between 15.5 and 32.3 Å were used to create all distribution plots.

### Creating the new model.

The full Ca^2+^ saturated structure consistent with the Yamada et al. structure was created by initially aligning the 30 actin monomers from our structure to the Yamada et al. structure, which contains 15 actin monomers (35). The cTnC proteins from our structure were translated to align with Yamada et al.’s, and the structural Ca^2+^ ions were added into the respective binding pockets. The full cTnI proteins from our structure (residues 1–210) were aligned to fit the Yamada et al. cTnI fragments (residues 41–166). The structured portions of the cTnI proteins aligned well; however, the unstructured terminal ends clashed with Tm and actin. To address the clashing, dihedral angles of a couple of residues in the unstructured regions were rotated until cTnI no longer clashed with other proteins. To align cTNT, the protein was initially split into 2 fragments (residues 1–198 and 199–288) so that each individual fragment could be aligned to the 2 fragments in the Yamada et al. structure (residues 99–150 and 199–272). Once the individual fragments were aligned, the 2 fragments were stitched together in VMD and minimized in NAMD so that the bond connecting them, located in the unstructured cTnT1-cTnT2 linker region, was an acceptable length. Dihedral angles of a couple of residues in the unstructured N- and C-termini of cTnT were rotated to eliminate any clashing of atoms that occurred after alignment. In the Yamada et al. structure, the C-terminus side of Tm in the overlap region contained residues 11 to 284, while the N-terminus side contained residues –1 to 29. The Tm structure Yamada et al. used to fit their cryo-EM data only stretched across 6 actin monomers, not 7 monomers, suggesting an incorrect helical pitch, also noted in previously published structures (56). Therefore, to shift our Tm coils into the new location agreeing with Yamada et al., collective variables defined as distance were used in NAMD to constrain segments of the center of mass (COM) of our Tm dimers to the COM of the dimers in the Yamada et al. structure (54). This allowed for the correct placement of Tm in the overlap region with respect to actin and cTnT, while maintaining the correct helical pitch in our structure. Once one side of dimers was aligned correctly, they were replicated to the other side of the CTF. Finally, an extra actin monomer with its respective ADP and calcium ion was added to the end of the structure to prevent the ends of the shifted Tm chains from hanging off the end of the structure. The final structure agreed with previously published refined structures, validating the structure used in this study (36, 56). Once all proteins were aligned correctly, the structure was minimized and heated in implicit solvent and then equilibrated in explicit solvent in NAMD using the same methods previously described. Mutation building and MD simulation parameters were performed using the same methods described above, except the overlap region average spanned between cTnT residues 90 and 130 because of shifting of the helices in the new model.

### Thermal stability and cooperativity of unfolding of the WT and mutated CTF.

The heat capacity (kJ/mol*K) of WT or mutant (D20N-Tm, S215L-Tm, R92Q-cTnT, or Δ96E-cTnT) CTF (Actin/Tm/Tn in a 3:2:2 reconstitution ratio) was measured via DSC (1°C/min heat rate from 20°C to 70°C) and graphed as a function of temperature. All experiments were performed in a HEPES buffer containing 30 mM HEPES, 150 mM KCl, 1 mM MgCl_2_, 200 μM NaATP, 200 μM CaCl_2_, and 1 mM β-ME. The complex raw thermogram generated contains information on 5 distinct unfolding events (in order of denaturation temperature, low to high: C-terminal Tm, Tm-Tn array away from actin, N-terminal Tm, Tn complex, actin). The raw data were then fit with a model generated by using 4-gaussian distribution (excluding actin denaturation) following baseline buffer heat subtraction and normalization to total mass of reconstituted protein (kept at ~2 mg/mL, 7.9 μM Tn, 7.9 μM Tm, 18.4 μM actin) using NanoAnalyze software (TA Instruments). The thermal stability results and a raw trace containing the first 4 unfolding events (actin denaturation excluded) after fitting can be found in Supplemental Figure 10 and Supplemental Figure 11, respectively. Each denaturation event (above) includes information on the maximum heat capacity (melt temperature, Tm, aka thermal stability) of the protein and the FWHM of the peak. For these experiments, only the peak corresponding to the Tm-Tn denaturation from actin is reported because it is the most informative value for our purposes. We refer to this value as conformational flexibility (the inverse cooperativity of unfolding). An *n* = 3 to 4 individual reconstitutions was used.

### Statistics.

The mean and SEM were calculated using a bootstrapping method in R (57). Values were calculated using 1000 bootstraps with replacement assuming the underlying distribution of distances is asymmetric. Supplemental Tables 1 and 2 summarize the mean and SEM for each variant analyzed with respect to WT. The standardized values for each variable, or *z* score, were calculated for each variant in the respective region using

where *x* is the variable mean, is the average value in the region, and σ_x_ is the SD of the region. From the *z* scores, the Euclidean distance from the origin was calculated using

where *x* is the *z* score from the change in overlap distance, *y* is the *z* score from the change in actin-Tm distance, and *z* is the *z* score from the change in distance between N-terminal Tm coils.

For all DSC experiments, a 1-way ANOVA with Dunnett’s correction for multiple comparisons was used to compare each mutation with WT CTF using Prism version 9.0.0 (GraphPad). *P* < 0.05 was considered statistically significant: * *P* < 0.05 versus WT CTF, ** *P* < 0.01 versus WT, *** *P* < 0.005 versus WT, **** *P* < 0.0001 versus WT. Absolute *P* values can be found in Supplemental Table 4.

### Study approval.

This study did not involve human or animal participants and qualified for exempt review by the Institutional Review Board of the University of Arizona.

## Author contributions

ABM and APB performed the in silico modeling under the supervision of SDS. ABM analyzed computational results and determined reclassifications. AED purified all proteins. MLL performed all DSC experiments. ABM, APB, MLL, JCT, and SDS wrote the manuscript.

## Supplementary Material

Supplemental data

## Figures and Tables

**Table 1:**
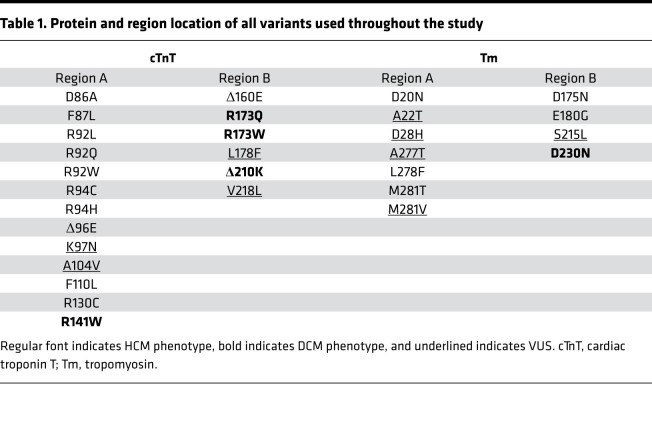
Protein and region location of all variants used throughout the study

**Table 2:**
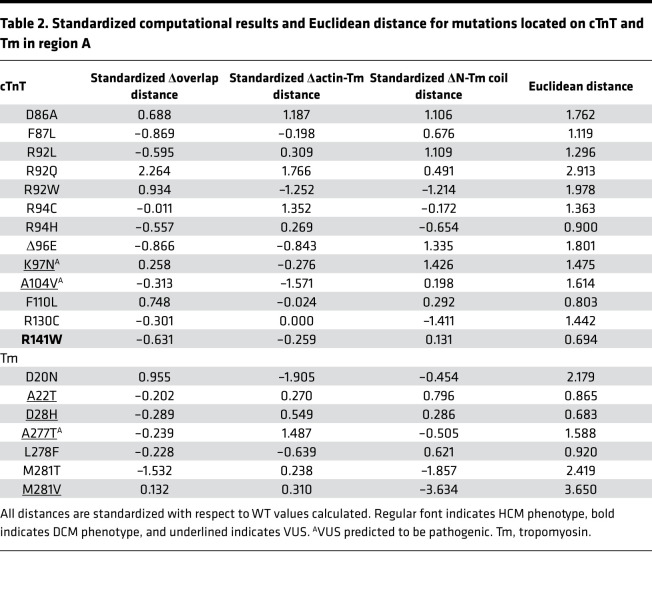
Standardized computational results and Euclidean distance for mutations located on cTnT and Tm in region A

**Table 3:**
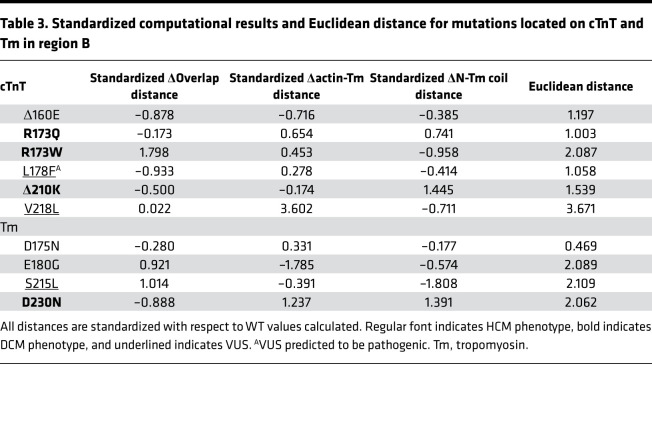
Standardized computational results and Euclidean distance for mutations located on cTnT and Tm in region B

**Table 4:**
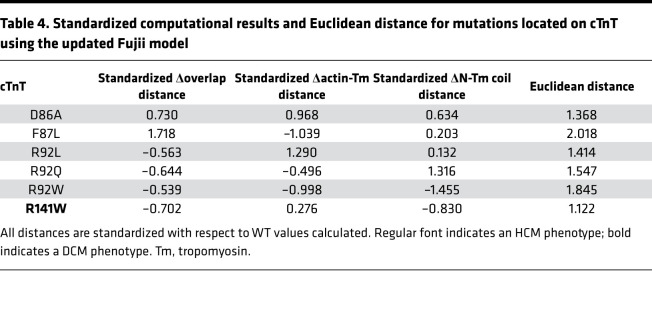
Standardized computational results and Euclidean distance for mutations located on cTnT using the updated Fujii model

**Table 5:**
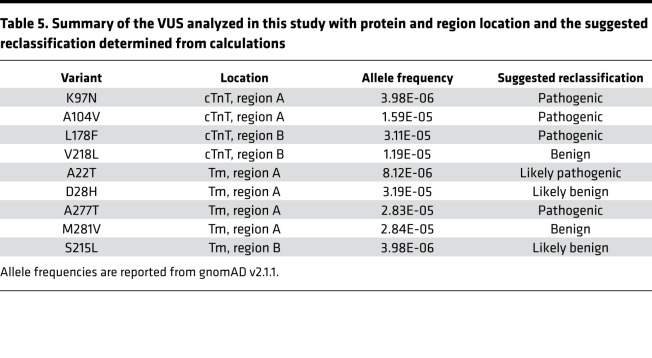
Summary of the VUS analyzed in this study with protein and region location and the suggested reclassification determined from calculations

## Version 1 10/26/2021

In-Press Preview

## Version 2 12/08/2021

Electronic publication
