# Supplementary material for: Computational and biophysical determination of pathogenicity of variants of unknown significance in cardiac thin filament
Source: JCI Insight. 2021 Dec 8;6(23):e154350. doi: 10.1172/jci.insight.154350 (PMC8675185; doi:10.1172/jci.insight.154350)
Supplement: Supplemental data [file jciinsight-6-154350-s099.pdf]

Supplemental Figures

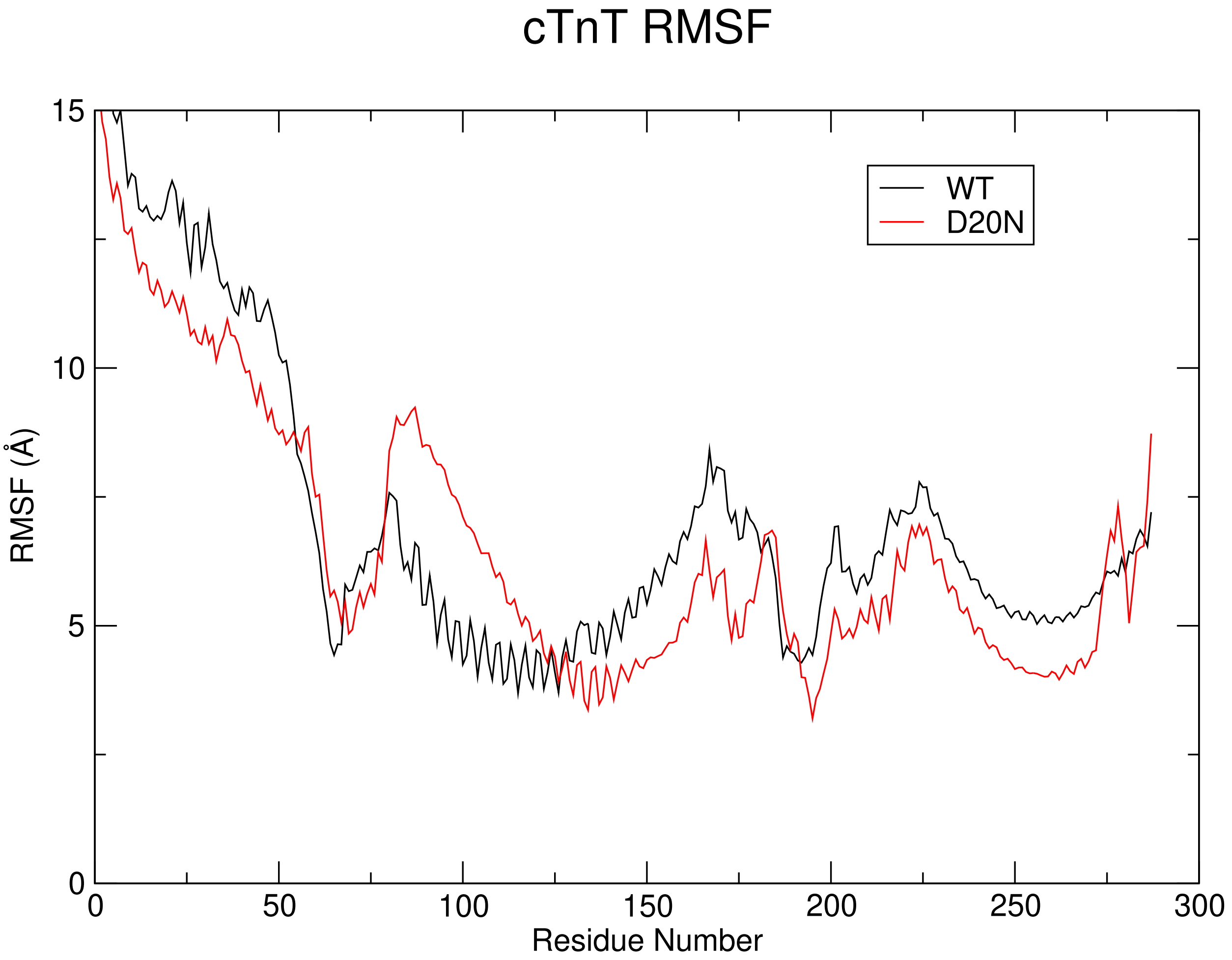

Supplemental Figure 1. RMSF of cTnT for wildtype (black) and the Tm mutation, D20N. Residue number indicates the alpha carbon for each residue and the relative location on the cTnT chain. Plots are linear representations of the entire protein showing the fluctuations of all alpha carbons with respect to the average location.

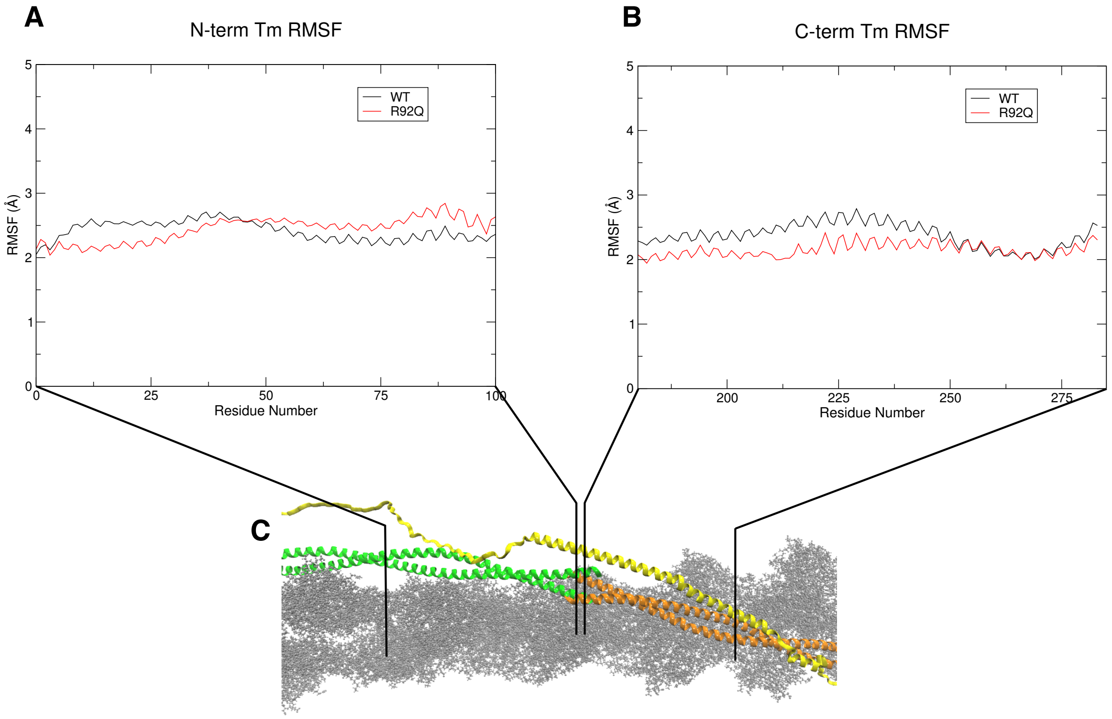

Supplemental Figure 2. Root Mean Squared Fluctuation (RMSF) plots for R92Q$_{cTnT}$ (red) and wildtype (black) for the N-terminus region of Tm (A) and the C-terminus region of Tm (B). Corresponding regions on the model for each plot are shown in C. Residue number indicates the alpha carbon for each residue and the relative location on the Tm chain. Plots are linear representations of the entire protein showing the fluctuations of all alpha carbons with respect to their average location.

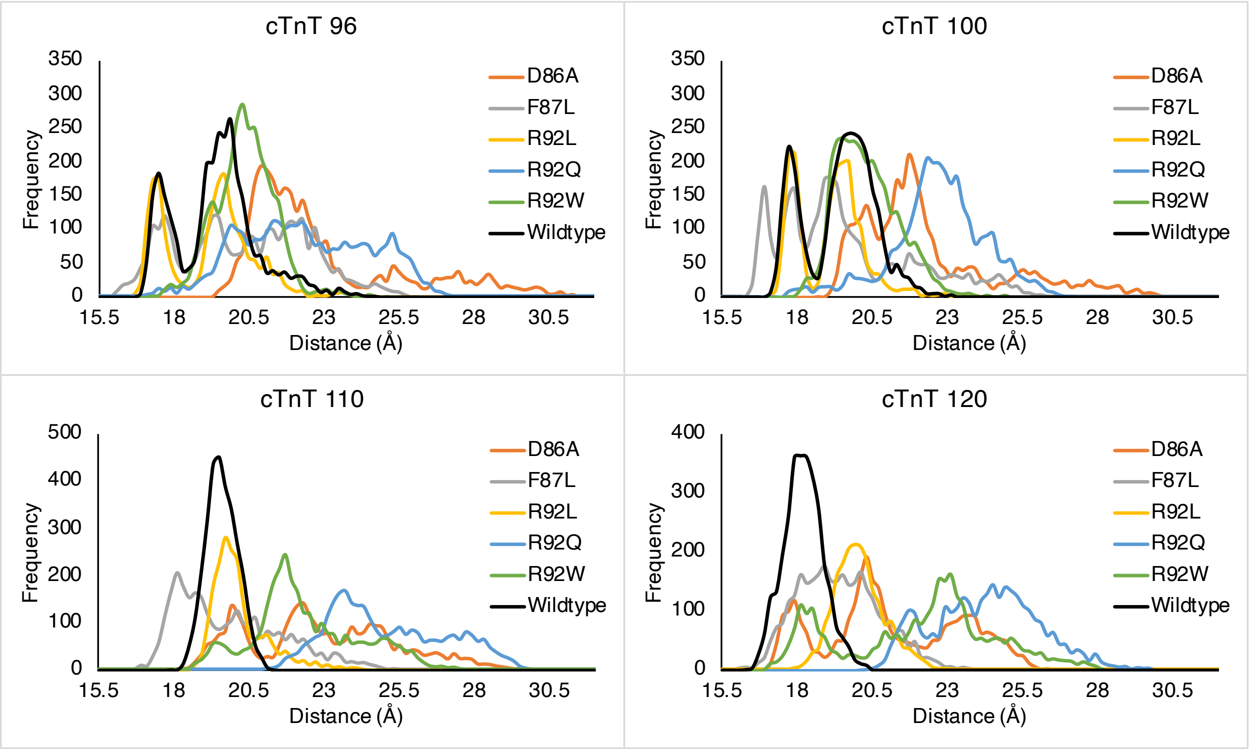

Supplemental Figure 3. Binned distributions of distances across all trajectories between the specified cTnT residue and the two Tm chains for wildtype and variants on cTnT in region A (D86A, F87L, R92L, R92Q, R92W). These distances were incorporated in the overall average distance between cTnT and Tm in the overlap region.

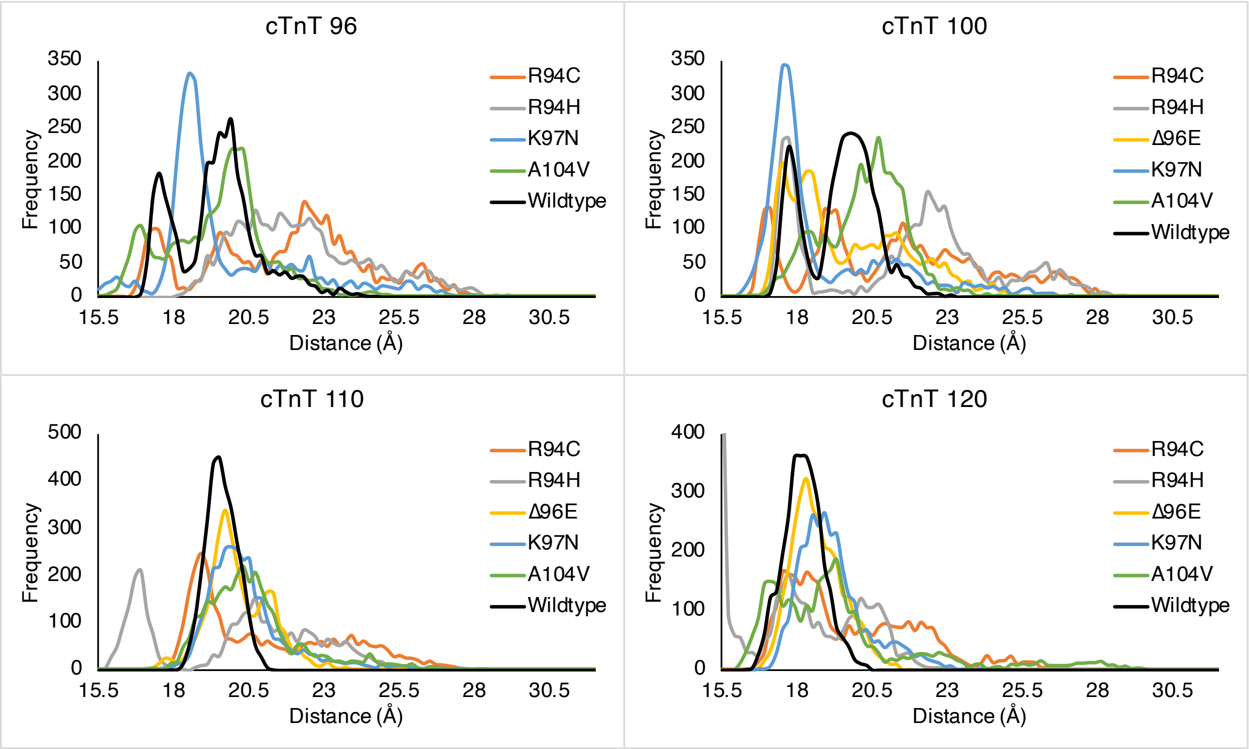

Supplemental Figure 4. Binned distributions of distances across all trajectories between the specified cTnT residue and the two Tm chains for wildtype and variants on cTnT in region A (R94C, R94H, Δ96E, K97N, A104V). These distances were incorporated in the overall average distance between cTnT and Tm in the overlap region.

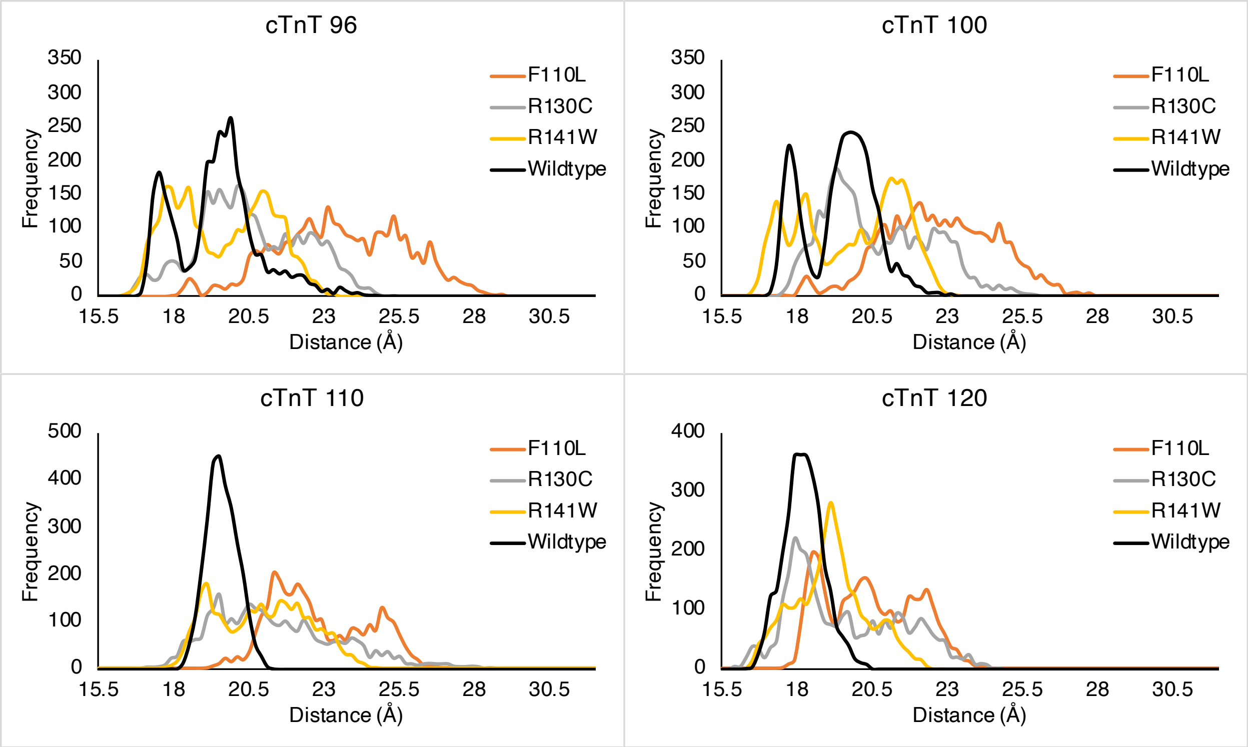

Supplemental Figure 5. Binned distributions of distances across all trajectories between the specified cTnT residue and the two Tm chains for wildtype and variants on cTnT in region A (F110L, R130C, R141W). These distances were incorporated in the overall average distance between cTnT and Tm in the overlap region.

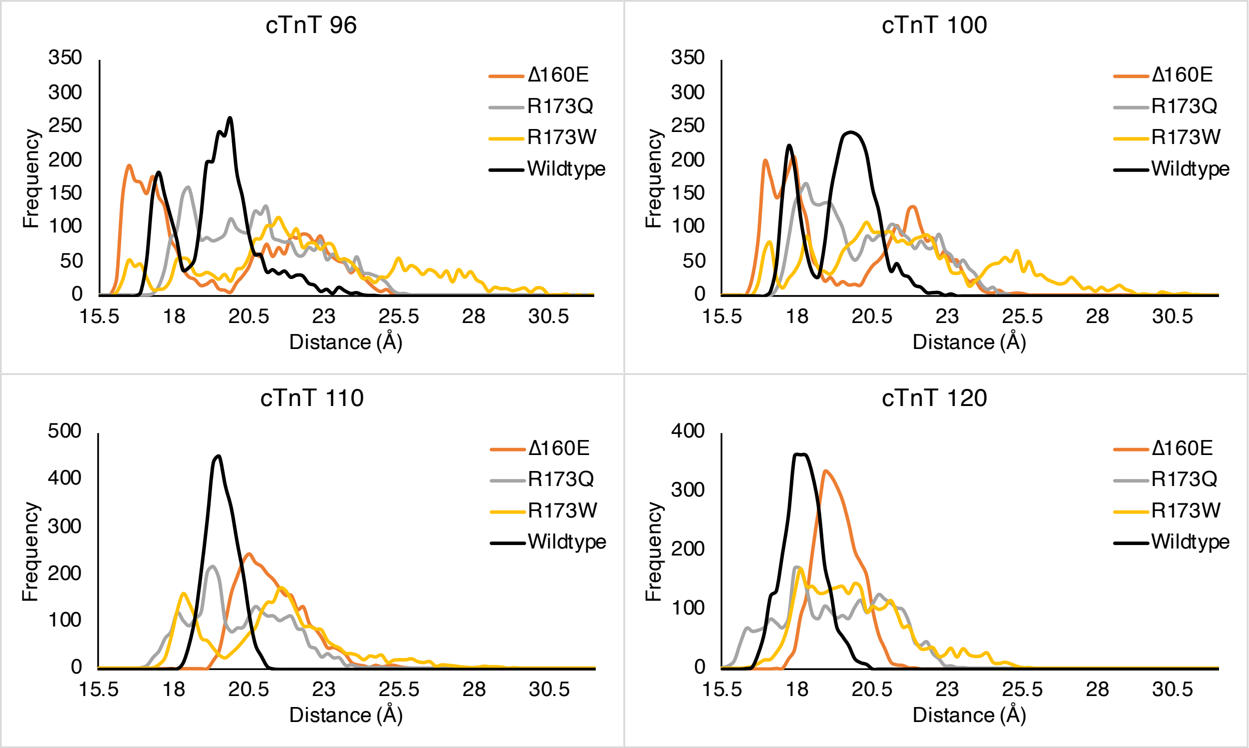

Supplemental Figure 6. Binned distributions of distances across all trajectories between the specified cTnT residue and the two Tm chains for wildtype and variants on cTnT in region B (Δ160E, R173Q, R173W). These distances were incorporated in the overall average distance between cTnT and Tm in the overlap region.

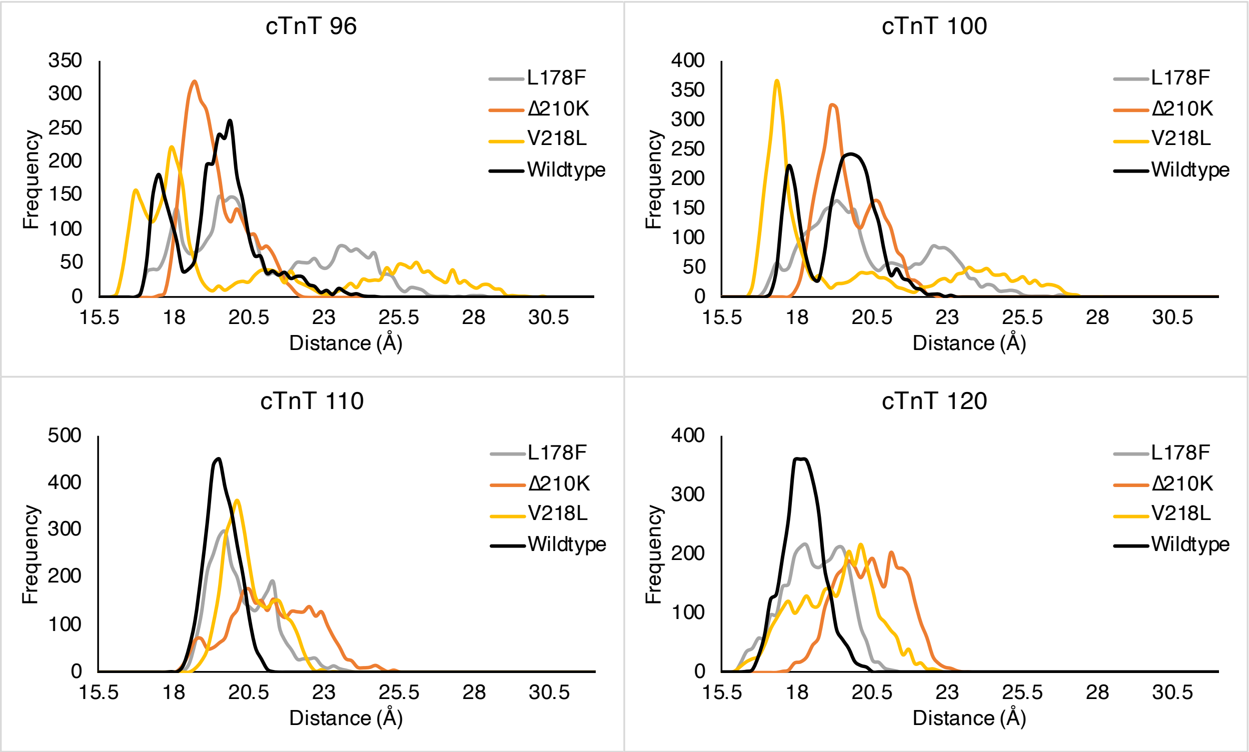

Supplemental Figure 7. Binned distributions of distances across all trajectories between the specified cTnT residue and the two Tm chains for wildtype and variants on cTnT in region B (L178F, Δ210K, V218L). These distances were incorporated in the overall average distance between cTnT and Tm in the overlap region.

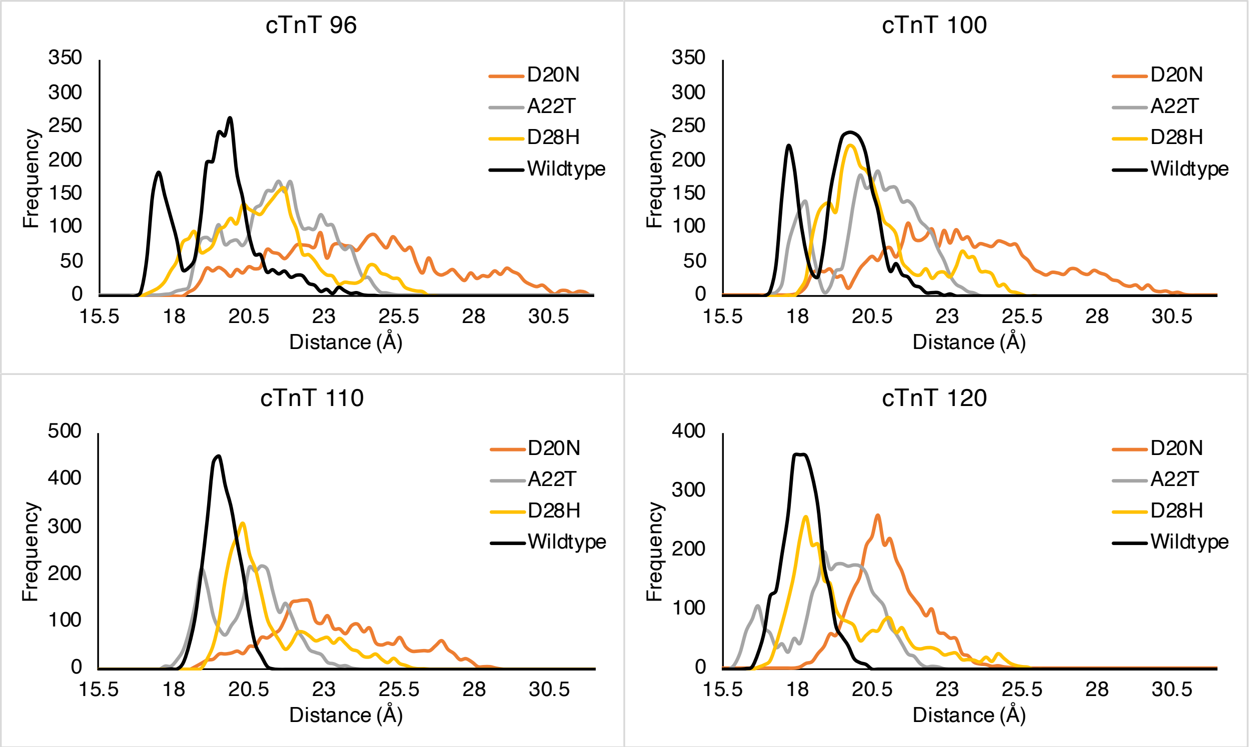

Supplemental Figure 8. Binned distributions of distances across all trajectories between the specified cTnT residue and the two Tm chains for wildtype and variants on Tm in region A (D20N, A22T, D28H). These distances were incorporated in the overall average distance between cTnT and Tm in the overlap region.

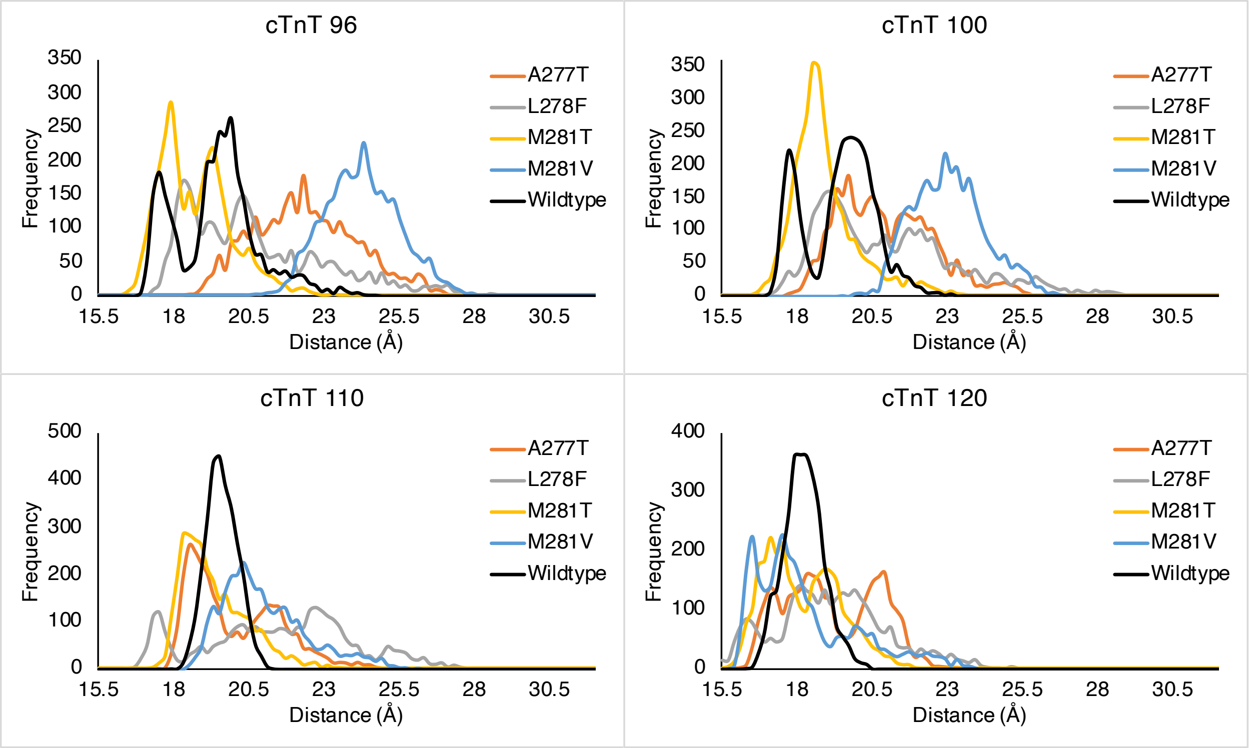

Supplemental Figure 9. Binned distributions of distances across all trajectories between the specified cTnT residue and the two Tm chains for wildtype and variants on Tm in region A (A277T, L278F, M281T, M281V). These distances were incorporated in the overall average distance between cTnT and Tm in the overlap region.

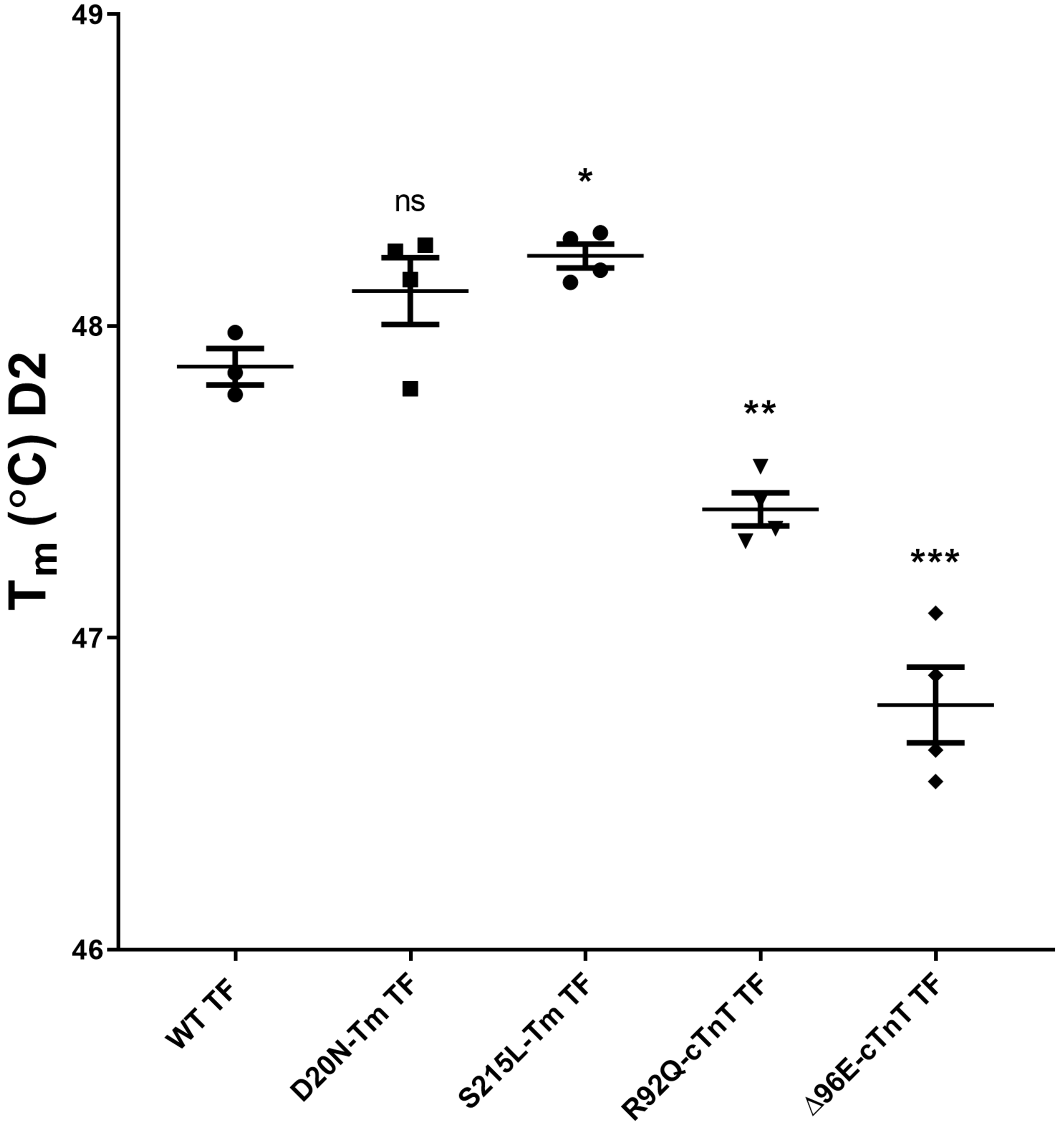

Supplemental Figure 10. Thermal stability results from differential scanning calorimetry experiments for fully reconstituted thin filaments of wildtype (WT), $D20N_{Tm}$, $S215L_{Tm}$, $R92Q_{cTnT}$, and $\Delta96E_{cTnT}$. Sample sizes of n=4 for WT and each mutant. The error bars represent mean ± SEM. Statistical analysis was performed via one-way ANOVA with Dunnett correction for multiple comparison using Prism version 9.0.0 (GraphPad, San Diego, CA). Significance is defined by $p < 0.05$ vs WT thin filament (*), $p < 0.01$ vs WT (**), $p < 0.005$ vs WT (***), $p < 0.0001$ vs WT (****).

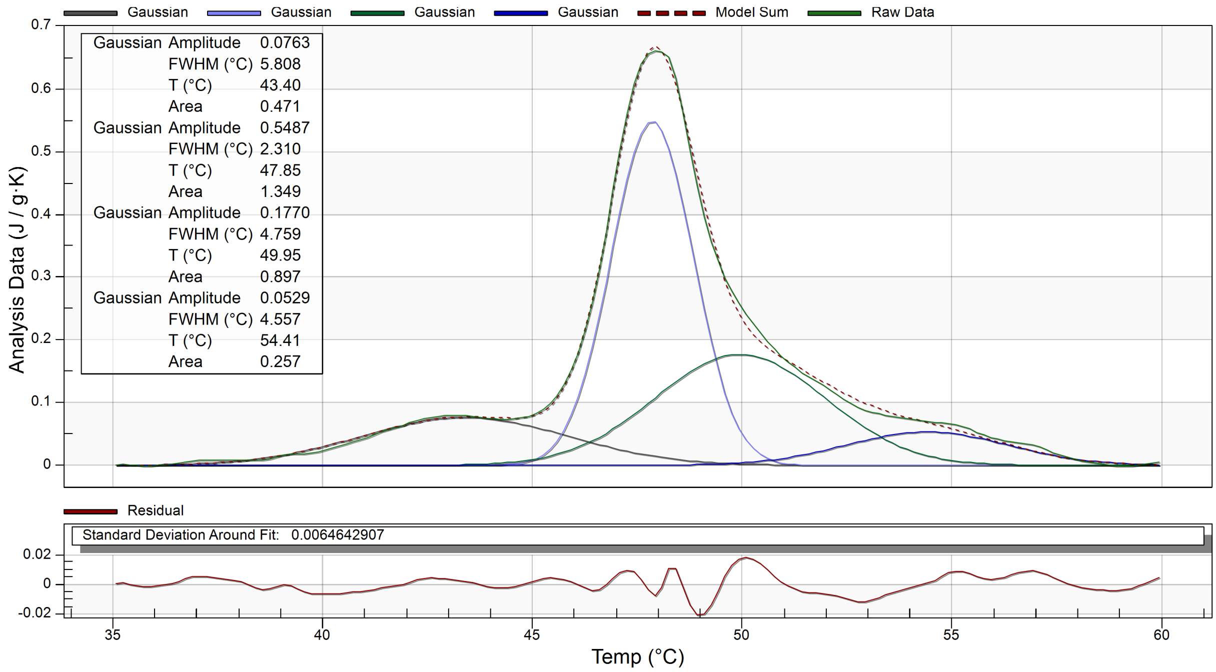

Supplemental Figure 11. A raw trace containing the first 4 unfolding events (actin denaturation excluded) post baseline subtraction and normalization to total protein mass. The raw data is represented by the solid green line, the model sum of the 4-Gaussian fit is represented by the red dashed line. The FWHM, Tm (T ℃), Amplitude, and area of each individual Gaussian from this single run are summarized in the inset table. These fit parameters are not constrained, but allowed to vary during fitting. For our purposes, only the values associated with the second Gaussian (Tm-Tn array denaturation from actin) are reported. A plot residual trace with standard deviation around the fit is shown beneath the trace.

Supplemental Tables

Supplemental Table 1. Computational results for variants located on cTnT and Tm in region A. All distances are reported as changes with respect to the wildtype values calculated ($\bar{x} \pm S.E$). Regular font indicates a HCM phenotype, bold indicates a DCM phenotype, and italicized and underlined indicates a variant of unknown significance.

Variant	ΔOverlap Distance (Å)	ΔActin-Tm Distance (Å)	ΔN-term Tm Coil Distance (Å)
cTnT			
D86A	2.15 ± 0.01	-0.02 ± 0.02	0.41 ± 0.01
F87L	0.21 ± 0.01	-0.27 ± 0.02	0.32 ± 0.01
R92L	0.55 ± 0.01	-0.18 ± 0.02	0.41 ± 0.01
R92Q	4.12 ± 0.01	0.09 ± 0.02	0.28 ± 0.01
R92W	2.46 ± 0.01	-0.46 ± 0.02	-0.10 ± 0.01
R94C	1.28 ± 0.01	0.01 ± 0.02	0.13 ± 0.01
R94H	0.60 ± 0.01	-0.19 ± 0.02	0.02 ± 0.01
Δ96E	0.22 ± 0.01	-0.39 ± 0.02	0.46 ± 0.01
K97N	1.62 ± 0.01	-0.29 ± 0.02	0.48 ± 0.01
A104V	0.91 ± 0.01	-0.52 ± 0.02	0.21 ± 0.01
F110L	2.23 ± 0.01	-0.24 ± 0.02	0.23 ± 0.01
R130C	0.92 ± 0.01	-0.24 ± 0.02	-0.14 ± 0.01
**R141W**	0.51 ± 0.01	-0.28 ± 0.02	0.20 ± 0.01
Tm			

D20N	2.49 ± 0.01	-0.58 ± 0.02	0.07 ± 0.01
A22T	1.04 ± 0.01	-0.19 ± 0.02	0.34 ± 0.01
D28H	0.94 ± 0.01	-0.13 ± 0.02	0.23 ± 0.01
A277T	1.00 ± 0.01	0.04 ± 0.02	0.06 ± 0.01
L278F	1.01 ± 0.01	-0.35 ± 0.02	0.31 ± 0.01
M281T	-0.61 ± 0.01	-0.19 ± 0.02	-0.24 ± 0.01
M281V	1.46 ± 0.01	-0.18 ± 0.02	-0.63 ± 0.01

Supplemental Table 2. Computational results for variants located on cTnT and Tm in region B. All distances are reported as changes with respect to the wildtype values calculated ($\bar{x} \pm S.E$). Regular font indicates a HCM phenotype, bold indicates a DCM phenotype, and italicized and underlined indicates a variant of unknown significance.

Variant	ΔOverlap Distance (Å)	ΔActin-Tm Distance (Å)	ΔN-term Tm Coil Distance (Å)
cTnT			
Δ160E	0.41 ± 0.01	-0.40 ± 0.02	0.22 ± 0.01
**R173Q**	0.78 ± 0.01	-0.26 ± 0.02	0.14 ± 0.01
**R173W**	1.81 ± 0.01	-0.28 ± 0.02	0.09 ± 0.01
L178F	0.38 ± 0.01	-0.30 ± 0.02	0.12 ± 0.01
**Δ210K**	0.61 ± 0.01	-0.34 ± 0.02	0.65 ± 0.01
V218L	0.88 ± 0.01	0.02 ± 0.02	0.14 ± 0.01
Tm			
D175N	0.72 ± 0.01	-0.30 ± 0.02	0.27 ± 0.01
E180G	1.35 ± 0.01	-0.50 ± 0.02	0.18 ± 0.01
S215L	1.40 ± 0.01	-0.36 ± 0.02	-0.12 ± 0.01
**D230N**	0.41 ± 0.01	-0.21 ± 0.02	0.64 ± 0.01

Supplemental Table 3. Computational results for variants located on cTnT in region A using the new Fujii model. All distances are reported as changes with respect to the wildtype values calculated ($\bar{x} \pm S.E$). Regular font indicates a HCM phenotype and bold indicates a DCM phenotype.

cTnT Variant	ΔOverlap Distance (Å)	ΔActin-Tm Distance (Å)	ΔN-term Tm Coil Distance (Å)
D86A	1.62 ± 0.01	1.43 ± 0.04	0.09 ± 0.01
F87L	2.44 ± 0.01	-0.29 ± 0.04	0.02 ± 0.01
R92L	0.55 ± 0.01	1.71 ± 0.04	0.01 ± 0.01
R92Q	0.48 ± 0.02	0.18 ± 0.04	0.20 ± 0.01
R92W	0.57 ± 0.01	-0.25 ± 0.04	-0.25 ± 0.01
**R141W**	0.44 ± 0.01	0.84 ± 0.04	-0.15 ± 0.01

Supplemental Table 4. Absolute p values for each mutation compared to wildtype thin filament for differential scanning calorimetry experiments.

Cardiac TF Mutation (vs WT TF)	T_m (°C) Adjusted P Value	FWHM (°C) Adjusted P Value
D20N-Tm	0.2072	0.0002
S215L-Tm	0.0436	<0.0001
R92Q-cTnT	0.0094	0.0248
Δ96E-cTnT	<0.0001	0.0093
